# Long-Term Outcome Following Treatment With Allogeneic Mesenchymal Stem/Stromal Cells for Radiation-Induced Hyposalivation and Xerostomia

**DOI:** 10.1093/stcltm/szae017

**Published:** 2024-06-10

**Authors:** Kathrine Kronberg Jakobsen, Charlotte Duch Lynggaard, Natasja Paaske, Amanda-Louise Fenger Carlander, Jens Kastrup, Anne Werner Hauge, Robin Christensen, Christian Grønhøj, Christian von Buchwald

**Affiliations:** Department of Otorhinolaryngology, Head and Neck Surgery and Audiology, Rigshospitalet—Copenhagen University Hospital, Copenhagen, Denmark; Section for Biostatistics and Evidence-Based Research, The Parker Institute, Bispebjerg and Frederiksberg Hospital, Copenhagen, Denmark; Department of Otorhinolaryngology, Head and Neck Surgery and Audiology, Rigshospitalet—Copenhagen University Hospital, Copenhagen, Denmark; Department of Otorhinolaryngology, Head and Neck Surgery and Audiology, Rigshospitalet—Copenhagen University Hospital, Copenhagen, Denmark; Department of Otorhinolaryngology, Head and Neck Surgery and Audiology, Rigshospitalet—Copenhagen University Hospital, Copenhagen, Denmark; Cardiology Stem Cell Centre, The Heart Centre, Rigshospitalet, Copenhagen, Denmark; Department of Clinical Immunology, Rigshospitalet, Copenhagen, Denmark; Section for Biostatistics and Evidence-Based Research, The Parker Institute, Bispebjerg and Frederiksberg Hospital, Copenhagen, Denmark; Research Unit of Rheumatology, Department of Clinical Research, University of Southern Denmark, Odense University Hospital, Odense, Denmark; Department of Otorhinolaryngology, Head and Neck Surgery and Audiology, Rigshospitalet—Copenhagen University Hospital, Copenhagen, Denmark; Department of Otorhinolaryngology, Head and Neck Surgery and Audiology, Rigshospitalet—Copenhagen University Hospital, Copenhagen, Denmark

**Keywords:** mesenchymal stem/stromal cells, adipose-derived mesenchymal stem/stromal cells, xerostomia, hyposalivation, radiotherapy

## Abstract

**Background:**

Adipose-derived mesenchymal stem/stromal cells (ASCs) are proposed as a new xerostomia treatment. The study evaluated the long-term safety and effectiveness of allogeneic ASCs in radiation-induced xerostomia among patients with previous oropharyngeal cancer.

**Methods:**

This study constitutes 3-year follow-up on the original 10 patients who received allogeneic ASCs injections to the submandibular and parotid glands as part of the MESRIX-II trial. The MESRIX-II trial included the preliminary 4-month follow-up. The primary endpoint was long-term safety. Secondary endpoints were effectiveness evaluated by changes in salivary flow rate and patient-reported outcomes (PROs). Immune response was evaluated by assessing the development of donor-specific antibodies (DSA).

**Findings:**

All 10 MESRIX-II patients completed the long-term follow-up (ie, no missing data). During the long-term follow-up, 2 patients encountered a significant adverse event, which was determined to be unrelated to the treatment. No DSAs were detectable at 3 years. The stimulated salivary flow rate increased significantly from an average of 0.66 mL/minute at baseline to 0.86 mL/minute at follow-up, corresponding to an increase of 0.20 [95% CI 0.08 to 0.30] mL/minute, or approximately 30%. Among the PROs, sticky saliva symptoms were reduced, with a −20.0 [95% CI −37.3 to −2.7] units.

**Interpretation:**

In conclusion, this study is the first to present long-term follow-up outcomes of allogeneic ASC treatment as a therapeutic option for radiation-induced xerostomia. The study found that ASC treatment appears safe, and there were no indications of adverse immune responses at the 3-year follow-up. Further studies are warranted to evaluate the findings in larger settings.

## Graphical Abstract

**Graphical Abstract:**
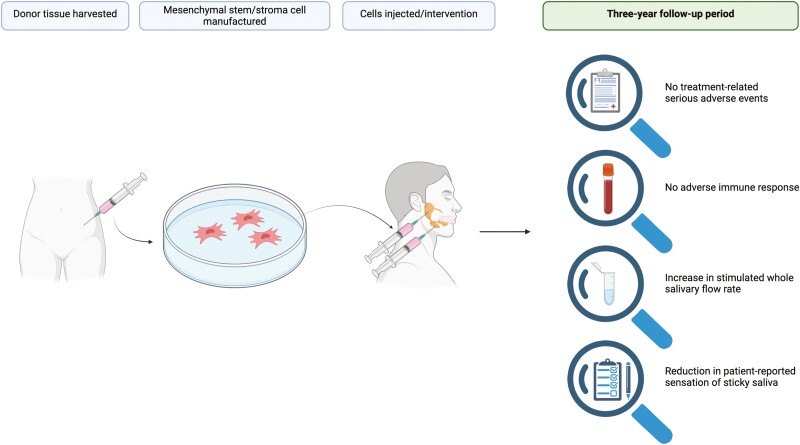

**Lessons Learned**

- The administration of allogeneic ASCs to the 4 major salivary glands was deemed safe, with no patients experiencing treatment-related serious adverse events throughout the 3-year follow-up period.
- At the 3-year follow-up, none of the patients exhibited an immunological response to the ASC treatment.
- ASC treatment led to a notable increase in stimulated salivary flow rate at the 3-year follow-up.

**Significance Statement**

Effective treatment for radiation-induced xerostomia in head and neck cancer survivors is scarce, and there is a lack of long-term data on allogeneic adipose-derived mesenchymal stem/stromal cell (ASC) therapy. This study assessed the 3-year safety and efficacy of ASC injections to the salivary glands. The results highlighted ASC treatment’s safety, with no treatment-related serious adverse events during the long-term follow-up. Immune reaction was temporary, with no donor-specific antibodies observed after 3 years. Promising clinical effectiveness was also indicated, emphasizing the need for more extensive research to enhance our understanding and optimize ASC treatments.

## Background

Head and neck cancer represents a significant global health concern, with over 900,000 new cases annually.^1^ The most common side effect of head and neck cancer treatment is hyposalivation and xerostomia, dry mouth syndrome, due to radiation-induced damage of the salivary glands.^2^ Xerostomia is a debilitating condition that impacts both the health and quality of life (QoL) of affected patients.^3^ Currently, the available treatments for xerostomia focus primarily on symptomatic relief, underscoring the need for novel and more effective therapeutic strategies.^4-7^

In 2015, our research group conducted a randomized, placebo-controlled study involving 30 patients investigating intraglandular injections to the submandibular glands of autologous adipose-derived mesenchymal stem/stromal cell (ASC) as a treatment option for restoring damaged salivary glands. Treatment with ASCs was safe and resulted in a significant increase in saliva production.^8,9^ Our long-term follow-up of patients treated with autologous ASCs demonstrated that the treatment was safe and had a positive impact on xerostomia symptoms.^10^ However, autologous ASC production yielded technical difficulties, and the process was inconvenient for the patients as they had to go through liposuction, prompting a shift to allogeneic ASCs. In 2019, our first-in-human study of allogeneic ASCs demonstrated early safety and potential clinical effect, with an increase in saliva production and a reduction in xerostomia-related symptoms after 4 months.^11^ However, there is a lack of long-term follow-up assessment for allogeneic ASC treatment.

The present study aimed to evaluate the long-term safety and effectiveness of allogeneic ASC injections in the major salivary glands for the treatment of radiation-induced xerostomia with a 3-year follow-up duration.

## Methods

### Study Design

The study was a single-center, open-label, phase I clinical trial aiming at investigating the long-term safety of intraglandular injections of ASC to the submandibular and parotid glands as a treatment for radiation-induced xerostomia in patients with prior oropharyngeal cancer. The patients were followed for 3 years. The study received approval from the National Ethics Committee (Protocol number: 1808924) and the Danish Medical Agency (EudraCT: 2018-003856-19). The study protocol was registered at the ClinicalTrials.gov database (NCT03874572) and complies with the Declaration of Helsinki. All included patients provided written consent.

### Study Setting

The study was conducted at the Department of Otorhinolaryngology, Head and Neck Surgery & Audiology, Rigshospitalet, Denmark. Data included in this study represent the long-term follow-up of patients included in the MESRIX-II trial.^11^ The investigator (K.K.J.) performed the long-term follow-up and was blinded to the baseline values of all patients.

### Patients

Patients included in the MESRIX-II trial^11^ were invited to participate in this long-term follow-up study (Supplementary 1 for original inclusion criteria). Each patient had initially received intraglandular injection of ASC to the parotid glands (50 million ASC in each) and the submandibular glands (25 million ASC in each). The ASC product was provided by the Cardiology Stem Cell Centre (CSCC)—Rigshospitalet, and produced as previously described.^12,13^

### Outcome Measures

#### Safety

Safety was evaluated by the development of serious adverse events (SAEs). The primary objective was to assess the long-term safety of intraglandular ASC treatment. Adverse events were graded according to the Common Terminology Criteria for Adverse Events v 5.0 (CTCAE). An SAE was defined as a CTCAE of grade 3 or more, equivalent to a medical condition necessitating hospitalization or an extended hospital stay, having life-threatening consequences requiring urgent intervention, or resulting in death. Data on safety were obtained through electronic medical files, patient reports, and patient examination.

#### Saliva Production

The secondary objectives were to evaluate the long-term effectiveness of intraglandular ASC treatment on saliva production. The saliva production was evaluated by sialometry measuring unstimulated whole salivary flow rate (UWS) and stimulated whole salivary flow rate (SWS). Additionally, patient-reported outcomes (PROs) of xerostomia were assessed using 2 questionnaires: the European Organization for Research and Treatment of Cancer Quality of Life Questionnaire, Head and Neck Module (EORTC QLQ-H&N35) and the Xerostomia Questionnaire (XQ). The EORTC QLQ-H&N35 was used to evaluate domains for dry mouth (HNDR), sticky saliva (HNSS), and swallowing (HNSW). Further, immunologic responses to ASC were assed with measurements of de novo human leukocyte antigen (HLA) development, as previously described.^10^

### Statistical Methods

The sample size for the study was based on the availability and extent of data derived from the previous MESRIX-II study.^10^ A statistical analysis plan was completed (Supplementary 2) before evaluating the collected data. The analyses were prespecified to correspond to the intention-to-treat population. We used a (multilevel) repeated-measures mixed-effects model with participants as a random effects factor and the particular outcome variable as the dependent variable. The time (6 levels: 0, 1, 5, 30, 120, and 1095 days) was set as a fixed effect factor based on a restricted maximum likelihood model. All 95% CIs and *P* values were 2-sided. All statistical analyses were performed in SAS- and R-studio.

## Findings

### Patients

From May 4, 2022 to January 10, 2023, the 10 patients included in MESRIX-II^10^ were invited to a long-term follow-up visit 3 years after ASC treatment. All patients accepted the invitation, and no dropouts occurred (Supplementary Fig. S1, flow diagram). Throughout the 3-year follow-up period, no protocol deviations were observed. For baseline characteristics of the included patient, see Supplementary Table S1.

### Safety

As reported in Table 1, no patients died during the study period. Two patients experienced an SAE during the long-term follow-up: 9 months after receiving ASC treatment, one patient was admitted to the hospital for a 3-day duration owing to a urinary infection. The patient received intravenous (i.v.) antibiotics. Subsequently, the same patient was hospitalized for severe pneumonia and pulmonary empyema, occurring 2.3 years post-ASC treatment. This hospitalization was extended for 39 days, including 15 days at the intensive care unit. As a part of the treatment, the patient received long-term i.v. antibiotics. Following this, the patient experienced 2 additional hospitalizations for pneumonia in the following 2 months, also necessitating i.v. antibiotics. In the last 6 months, the patient has been out of the hospital and recovered. Another (second) patient sustained a leg fracture following a fall 2.3 years after the ASC treatment. None of these SAEs were deemed related to the treatment. No patients experienced a new cancer or recurrence, and no patients developed any diseases of the salivary glands.

**Table 1.** Safety profile.

Adverse events	N	Sum (events)	Proportion (%)
Deaths, n (%)	10	0	0
SAEs, n (%)	10	2 (Patient 1 and 6)	20
TR-SAEs, n (%)	10	0	0

Abbreviation: TR-SAE, treatment-related serious adverse events.

### Salivary Flow Rate

**Figure 1.**
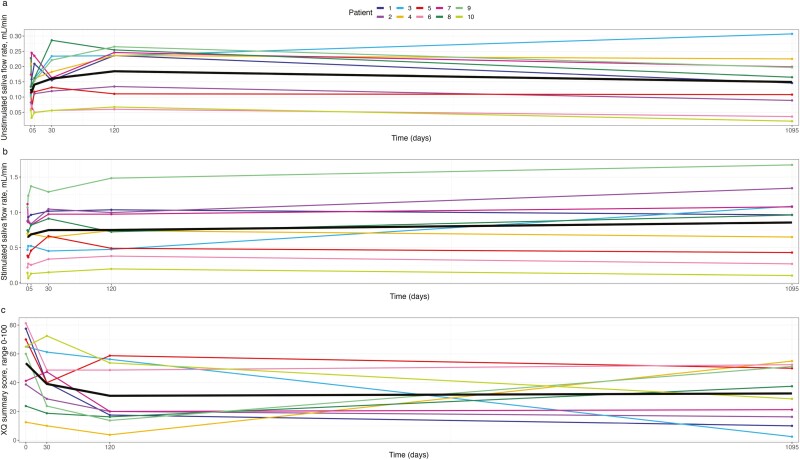
Change during the 3-year study period in (**A**) UWS, (**B**) SWS, and (**C**) PRO questionnaire XQ. Abbreviation: XQ, Xerostomia Questionnaire.

At the long-term follow-up visit, the least squares mean UWS was 0.15 mL/minute; this corresponded to a statistically nonsignificant increase of 0.02 [95% CI −0.01 to 0.06] mL/minute (Table 2) from baseline. Notably, the UWS initially increased from the baseline visit, with a UWS of 0.18 [95% CI 0.13 to 0.24] mL/minute at the 4-month follow-up visit. However, it subsequently showed a statistically nonsignificant decrease from 4 months to long-term follow-up (0.04 [95% CI −0.0007 to 0.07]; *P* = .054, Fig. 1a).

**Table 2.** Change from baseline to 3-year follow-up.

	Baseline (*n* = 10)	3 years (*n* = 10)	Difference [95% CI]	*P*-value
Change from baseline to 3 years				
Unstimulated saliva flow rate (mL/minute)	0.13 (0.02)	0.15 (0.02)	0.02 [−0.01 to 0.06]	.21
Unstimulated saliva flow rate (% )	0 (14.7)	18.4 (14.7)	18.4 [−11.7 to 48.4]	.22
Stimulated saliva flow rate (mL/minute)	0.66 (0.12.5)	0.86 (12.5)	0.20 [0.08 to 0.3]	.0022
Stimulated saliva flow rate (% )	0 (8.3)	27.7 (8.3)	27.7 [6.8 to 48.7]	.011
XQ summary score (0-100)	53.5 (6.6)	32.5 (6.6)	−21.0 [−37.5 to −4.5]	.015
EORCT QLQ-H&N35 (scores 0-100)				
HNDR	73.3 (7.6)	56.7 (7.6)	−16.7 [−38.5 to 5.1]	.13
HNSS	46.7 (8.3)	26.7 (8.3)	−20.0 [−37.3 to −2.7]	.025
HNSW	26.7 (5.2)	30.0 (5.2)	3.3 [−6.8 to 13.5]	.51

Estimates are reported as least squares means and SE unless otherwise indicated.

Abbreviations: EORTC QLQ-H&N35, the European Organization for Research and Treatment of Cancer Quality of Life Questionnaire, Head and Neck Module; HNDR, domains for dry mouth (HNDR); HNSS, domains for sticky saliva; HNSW, domains for swallowing; XQ, xerostomia Questionnaire.

The SWS increased significantly from 0.66 [95% CI 0.38 to 0.94] mL/minute at the baseline visit to 0.86 [95% CI 0.68 to 1.13] mL/minute at the long-term follow-up visit, corresponding to an increase of 27.7% [95% CI 6.8 to 48.7]. We thus observed a sustained increase in the SWS (Fig. 1b).

### Health-Related Quality of Life

The XQ summary score exhibited a significant decrease of −21.0 [95% CI −37.5 to −4.5] units from baseline to the 3-year follow-up period (Table 2). No statistically significant change was observed between the 4-month follow-up and the 3-year follow-up visit (*P* = .84, Fig. 1c). The dry mouth domain showed a statistically nonsignificant symptom decrease of −16.7 [95% CI −38.5 to 5.1] units from baseline to the 3-year follow-up (*P* = .13, Table 2). Sticky saliva exhibited a statistically significant symptom reduction of −20.0 [95% CI −37.3 to −2.7] units at the 3-year follow-up compared to baseline (*P* = .025, Table 2). The swallowing domain displayed no statistically significant change from baseline to 3-year follow-up (−3.3 [95% CI −13.5 to 6.8] units; *P* = .51, Table 2), and the initial significant decrease at 4-month follow-up was not sustained.

### Human Leukocyte Antigen

One patient exhibited preformed HLAs before the ASC treatment, and these antibodies remained detectable throughout all follow-up visits. Notably, the preformed antibodies for HLA class I initially showed an upregulation after the ASC treatment but gradually decreased to approximately pretreatment levels by the 3-year follow-up visit. Two patients developed donor-specific antibodies (DSAs) at 1-month post-treatment. However, these antibodies were no longer detectable at the 3-year follow-up. None of the patients developed DSAs for HLA class II or experienced an upregulation of preformed HLA class II after the treatment (Table 3).

**Table 3.** Human leukocyte antigen antibodies.

Patient	Donor	HLA	Baseline MFI	1-Month MFI	4-Month MFI	36-Month MFI
1	1	—	(−)	(−)	(−)	(−)
2	2	—	(−)	(−)	(−)	(−)
3	2	—	(−)	(−)	(−)	(−)
4	3	—	(−)	(−)	(−)	(−)
5	3	B60(40)	(−)	17 756	3470	(−)
6	1	A2	11 664	17 976	14 619	13 785
		Cw2	1515	4003	2329	1396
		DR4	4446	3853	5635	3088
7	2	—	(−)	(−)	(−)	(−)
8	1	—	(−)	(−)	(−)	(−)
9	2	—	(−)	(−)	(−)	(−)
10	1	A2	(−)	5056	11 242	(−)
		A24(9)	(−)	(−)	2381	(−)
		B44(12)	(−)	9813	10 175	(−)

Screening for HLA antibodies was performed on a luminex platform (Luminex 200 system or LABScan3D). Screening for HLA antibodies (IgG) was performed using the LABScreen Mixed assay (One Lambda) and if positive subsequent specification was performed using LABScreen Single Antigen assay (One Lambda). For LABScreen Single Antigen trimmed mean values were normalized for background and expressed as MFI. MFI > 1000 was defined as positive.

HLA typing for donor 1: HLA-A*02, 24; B*40, 44; C*02, 03; DRB1*01, 04; DQB1*03, 05.

HLA phenotype donor 1: HLA-A2, 24(9); B60(40), 44(12); Cw2, 10(3); DR1, 4; DQ5, 8(3).

HLA typing for donor 2: HLA-A*02, −; B*13, 40; C*03, 06; DRB1*07, 13; DQB1*02, 06.

HLA phenotype donor 2: HLA-A2, −; B13, 60(40); Cw6, 10(3); DR7, 13(6); DQ2, 61(1).

HLA typing for donor 3: HLA-A*01, −; B*08, 40; C*03, 07; DRB1*13, −; DQB1*06, −.

HLA phenotype donor 3: HLA-A1, −; B8, 60(40); Cw7, 10(3); DR13(6), −; DQ6(1), −.

Abbreviation: MFI, mean fluorescence intensity; (−): negative.

## Interpretation

This is the first study to present the long-term safety and effectiveness of allogeneic ASC injections to the major salivary glands as a treatment for radiation-induced xerostomia. To the best of our knowledge, this study offers the most extensive follow-up duration following allogeneic ASC treatment. Our findings demonstrate that treatment with ASC was safe, as no patients experienced treatment-related SAE during the long-term follow-up. Importantly, no patients developed salivary gland diseases, and none experienced a new cancer or a recurrence. Similar to previous findings, we thus confirmed the safety of intraglandular treatment with ASCs.^10,11^

Importantly, our study included both the immunological profile of the patients before the treatment and after the treatment. Two patients initially developed an HLA response after the ASC treatment. The HLA antibodies were no longer detectable at the 3-year follow-up, indicating a transient and self-resolving immune reaction. Mesenchymal stem cells have been considered hypoimmunogenic as they lack HLA class II expression.^14^ As we did not find any HLA class II antibodies, our study confirms the thesis that ASCs, indeed, are hypoimmunogenic, thereby reducing the risk of an immune response. One patient had preformed antibodies that initially were upregulated after the ASC treatment but reached pretreatment levels at the 3-year follow-up visit. This patient highlights the importance of measuring the immunological profile both before and after treatment. HLA antibodies are considered an unfavorable indicator of the effect of mesenchymal stem/stromal cells.^15^ For the 3 patients with HLA antibodies in our study, an improvement in PROs during the trial was reported. However, we did not find an effect of ASC treatment on the UWS for these patients. It is difficult to establish the clinical relevance of the observed immunologic change, but it is important to acknowledge potential concerns related to an HLA response, and there is a clear need for further studies with larger patient cohorts to address this matter adequately.

We initially observed an increase in the UWS 4 months after ASC injections.^11^ We did not find a statistically significant sustained increase in the UWS at the 3-year follow-up, prompting considerations about the potential necessity for repeated treatments. Several studies have demonstrated that repeated treatments with mesenchymal stem/stromal cells are more effective than a single treatment.^15-19^ Further studies investigating repeated treatments with intraglandular ASCs for a long-lasting effect are thus warranted.

The sustained increase in SWS observed at the long-term follow-up suggests a potential for longer-lasting effects within the parotid gland, as the parotid glands produce the majority of the SWS.^20^ It is worth noticing that 50 million ASCs were injected into the parotid glands contrary to the submandibular glands which each were treated with 25 million ASCs. This discrepancy in dosage was attributed to the larger size of the parotid glands compared to the submandibular glands. According to this study, a greater quantity of ASCs might lead to a more enduring effect. Further investigation into the mechanisms underlying this sustained increase is warranted to elucidate the potential long-term benefits of ASC treatment on salivary gland function.

A notable methodological strength of this study is Denmark’s public health care system, with treatments documented in electronic medical records, reducing recall bias. The investigator carrying out the long-term follow-up was blinded to the baseline and 4-month follow-up values. Further, no patients were lost to follow-up. The primary limitation of this study is the single-center, nonrandomized design, and small sample size. In addition, salivary gland scintigraphy was not performed during the long-term follow-up, given the absence of observable changes in the short-term follow-up.^10^ However, this decision raises the possibility that we might have overlooked certain aspects or potential developments. Additionally, we conducted the efficacy assessment only once at the long-term follow-up, but conducting sialometry multiple times would bolster the reliability and robustness, and could be considered for future studies.

In conclusion, this trial is the first study to present long-term follow-up outcomes of intraglandular allogeneic ASC treatment as a novel therapeutic option for radiation-induced xerostomia. The study encompassed a comprehensive 3-year follow-up period. Remarkably, no patients developed any treatment-related SAEs, and there were no indications of immune responses at the 3-year follow-up. Further, we found a significant increase in SWS. These findings provide valuable evidence supporting the safety of ASC treatment and support the hypothesis that ASCs may represent a promising new treatment option for xerostomia. Nonetheless, larger, blinded, placebo-controlled studies are necessary to provide more robust and unbiased evidence, building upon these findings and deepening our understanding of ASCs’ potential in managing radiation-induced xerostomia.

## Supplementary Material

Supplementary material is available at *Stem Cells Translational Medicine* online.

szae017_suppl_Supplementary_Table_Figure

## Data Availability

The data underlying this article will be shared on reasonable request to the corresponding author.

## Clinical Trials Information

The study protocol was registered at the ClinicalTrials.gov database (NCT03874572) and complies with the Declaration of Helsinki. All included patients provided written consent. The study was funded by the nonprofit Candys Foundation (grant 2017-218) and University Hospital of Copenhagen—Rigshospitalet (no grant number). The funders of the study had no role in the study design, data collection, data analysis, data interpretation, or writing of the manuscript.
