# Supplementary material for: Long-Term Outcome Following Treatment With Allogeneic Mesenchymal Stem/Stromal Cells for Radiation-Induced Hyposalivation and Xerostomia
Source: Stem Cells Transl Med. 2024 Jun 10;13(6):515–21. doi: 10.1093/stcltm/szae017 (PMC11165157; doi:10.1093/stcltm/szae017)
Supplement: szae017_suppl_Supplementary_Table_Figure [file szae017_suppl_supplementary_table_figure.docx]

Table of contents

[Supplementary 1 – Original inclusion criteria 2](#_Toc155339722)

[Supplementary 2 – Statistical Analysis Plan 3](#_Toc155339723)

[Supplementary Figure 1. Flow diagram illustrating the trial flow 10](#_Toc155339724)

[Reference 13](#_Toc155339725)

# **Supplementary 1 – Original inclusion criteria**

Inclusion criteria

1. Age: 18-75 years
2. Previous radiotherapy +/- chemotherapy for oropharyngeal cancer stage I- II (UICC-8, 2017).
3. 2 years follow-up without recurrence
4. Clinically reduced salivation and hyposalivation, evaluated by a screening (defined as an unstimulated salivary flow rate between 0.2mL/min and 0.05mL/min)
5. Grade 1-3 xerostomia
6. WHO Performance status 0-1

Exclusion criteria:

1. Any cancer in the previous 4 years (not including the head and neck cancer and basocellular carcinomas)
2. Xerogenic medications[1]
3. Penicillin or Streptomycin allergy
4. Any other diseases of the salivary glands, e.g. Sjogren’s syndrome or sialolithiasis
5. Previous parotid submandibular gland surgery
6. Previous treatment with any type of stem cells in the saliva glands
7. Breastfeeding, pregnancy, or planned pregnancy within the first two years after treatment
8. Smoking within the previous 6 months
9. Alcohol abuse (consumption must not exceed 7 units/week for women and 14 units/week for men (Danish National board health alcohol guidelines[2]))

# **Supplementary 2 – Statistical Analysis Plan**

**Title:**

Long-Term Outcomes Following Treatment with Allogeneic Mesenchymal Stromal Cell for Radiation-Induced Hyposalivation and Xerostomia in Previous Head and Neck Cancer Patients: Statistical Analysis Plan for the Long-Term MESRIX-II cohort study

**Trial registration:**

The National Ethics Committee protocol number: (Protocol number: 1808924)

The Danish Medical Agency (2018-000356-19)

ClinicalTrials.gov database (NCT03874572)

**SAP version:**

Version 1, 18^th^ of May 2023

SAP associated with protocol version 2.8

Primary Authors of the SAP: Kathrine Kronberg Jakobsen and Robin Christensen

**Roles and responsibilities:**

Senior biostatistician responsible: Robin Christensen, BSc, MSc, PhD; Professor

Principal investigator: Kathrine Kronberg Jakobsen, MD, PhD-fellow

Sponsor: Christian von Buchwald, MD, DMSc, professor.

This SAP is reported following the recommendations from “Guidelines for the Content of Statistical Analysis Plans in Clinical Trials.” by Gamble C, Krishan A, Stocken D, Lewis S, Juszczak E, Doré C, et al. published in JAMA 2017;318:2337-43.

**Introduction**

**Background and rationale**

Xerostomia, the subjective feeling of dry mouth, is a common side effect of radiotherapy after head and neck cancer. Intraglandular mesenchymal stem cell therapy has been studied as a potential treatment for xerostomia, with promising results. The MESRIX-II trial was designed as the first-in-human, phase 1b trial investigating ultrasound-guided allogeneic stem cell injections to the parotid glands and submandibular glands as a treatment for xerostomia. The study included 10 patients with xerostomia after radiotherapy for a previous Oropharyngeal Squamous Cell Carcinoma (OPSCC). The first part of the trial investigated the early trial outcomes and has previously been described[3]. In this long-term cohort study, we wish to investigate the long-term safety and late effect of allogeneic adipose stem cells (ASC) as a treatment for xerostomia based on the data collected as part of MESRIX-II.

**Objectives**

The primary safety objective was to descriptively report long-term safety. Secondary objectives were to evaluated change in unstimulated salivary gland function, stimulated salivary gland function, and patient-reported outcomes of xerostomia evaluated by the two questionnaires the Xerostomia Questionnaire (XQ), and the European Organisation for Research and Treatment of Cancer Quality of Life Questionnaire, Head and Neck-35 (EORTC QLQ-H&N35) from baseline to the three-years follow-up. Lastly, immune response to treatment with ASC, measured by the development of de novo human leucocyte antigen antibodies (HLA) as a response to ASC treatment in the first 3 years of the study period were evaluated.

**Study Methods**

**Trial design**

The study was designed as a single-centre, open-label, phase 1b cohort study. Study participants from the MESRIX-II trial will be invited for a clinical visit to assess the long-term safety and effect of allogeneic adipose tissue-derived mesenchymal stem cells (ASCs) as a treatment for radiation-induced hyposalivation and xerostomia.

**Randomisation**

NA

**Intervention Model:**

Single group Assignment

**Blinding**

NA

**Sample size and power considerations**

The sample size for the present spin-off study was not determined by formal statistical methods but was based on the extent and availability of data following the previous MESRIX-2 initiative (i.e., feasibility).

**Framework**

The study is an uncontrolled longitudinal observational study design.

**Statistical interim analyses and stopping guidance**

We have published the four months data [3].

**Timing of final analysis**

The primary endpoint, as well as the key secondary outcomes, will all be evaluated based on the up to three years assessment.

**Timing of outcome assessments**

Schedule of enrolment, interventions, and assessments.

|  | STUDY PERIOD | | | | | | |
| --- | --- | --- | --- | --- | --- | --- | --- |
|  | Enrolment | Allocation | Post-allocation | | | | Close-out |
| TIMEPOINT | *Up to 60 days prior to interventions* | 0 | *Follow-up 1 day after intervention* | *Follow-up 5 day after intervention* | *Follow-up 1 month after intervention* | *4 months after intervention* | *3 years after intervention* |
| ENROLMENT: |  |  |  |  |  |  |  |
| Eligibility screen | X |  |  |  |  |  |  |
| Informed consent | X |  |  |  |  |  |  |
| Allocation |  |  |  |  |  |  |  |
| INTERVENTIONS: |  |  |  |  |  |  |  |
| *ASC* |  | X |  |  |  |  |  |
| ASSESSMENTS: |  |  |  |  |  |  |  |
| *Safety* | X | X | X | X | X | X | X |
| *Sialometry* | X |  | X | X | X | X | X |
| *Quality of life questionnaires* | X | X |  |  | X | X | X |
| *Blood samples (HLA response)* | X |  |  |  | X | X | X |
| *Scintigraphy* | X |  | X |  |  | X |  |

**Statistical principles**

**Confidence intervals and P values**

The 95% confidence intervals and P values will be two-sided. We will not apply explicit adjustments for multiplicity, rather we will analyze and interpret these exploratory data analyses with caution. Thus, we will disclose that our exploratory findings might reveal a false discovery (i.e. possibly having a false-positive finding [rejecting a null hypothesis]).

**Adherence and protocol deviations**

We will define adherence as a participant who has a full registration and has completed every assessment for all the time points in the study. The adherence to the assessments will be summarised with number and percent compliance. All deviations from the protocol will be described.

**Analysis populations**

The primary analyses will be based on the Intention to Treat (ITT) population, i.e., based on the Full Analysis Set: The ITT principle asserts the effect of a treatment policy (that is, the planned treatment regimen), rather than the actual treatment given (i.e., it is independent of treatment adherence). Accordingly, participants allocated to the ASC treatment will be followed up, assessed, and analysed as members of the group, irrespective of their adherence to the planned course of treatment (i.e., independent of withdrawals and missing data in general).

We will use a (multilevel) repeated measures mixed effects model with participants as a random effects factor and the particular outcome variable (*Y*i) as a dependent variable. The time (days; 6 levels: 0, 1, 5, 30, 120, 1095) is set as a fixed effect factor based on a restricted maximum likelihood model. This statistical model holds all between-time comparisons for all assessment points up to 3 years from baseline/day 0, and allows for evaluation of the average changes, as well as the trajectory over time from baseline to 3 years follow-up.

**Trial population**

Screening data, eligibility, and recruitment

Information on screening, eligibility, and recruitment was published in our first paper [3].

**Baseline patient characteristics**

Descriptive statistics for categorical data will be evaluated as numbers and percentages while continuous data will be summarised by mean and standard deviation.

**Analysis**

**Outcome definitions**

*Primary outcome measure:*

Safety: evaluated by the number of patients with serious adverse events (incl. deaths) during the 3 years of the study observation period

*Secondary outcomes are the following measurements:*

1. Effectiveness: Saliva gland function measured as the change in unstimulated whole saliva flow rate. Timeframe: Up to 3 years.
   - The saliva flow rate will be measured as ml/min.
   - The saliva flow rate will be measured as percent change from baseline
2. Effectiveness: Saliva gland function measured as change in stimulated saliva flow rate. Timeframe: Up to 3 years.
   - The saliva flow rate will be measured as ml/min.
   - The saliva flow rate will be measured as percent change from baseline
3. Effectiveness: Impact on quality of life measured as a change in patient-reported outcome of quality of life and xerostomia. Patients will fill out the XQ. The results will be reported as a collected score.
4. Effectiveness: Impact on quality of life measured as a change in patient-reported outcome of quality of life and xerostomia. Patients will fill out the EORTC QLQ-H&N35.
   - HNDR
   - HNSS
   - HNSW
5. Immune response to treatment with ASC
   - Development of HLA as a response to ASC treatment in the first 3 years of the study period for each patient.

**Analysis methods**

The repeated measures designs aim to draw conclusions about the mean values of the populations from which participants are selected by considering treatment and time effects in the model. The objective is usually achieved by considering both treatment and time effects (as well as the interaction between them) in the model. Data will be analyzed using SAS, with the particular outcome variable (Yi) as the dependent variable, using a multilevel repeated measures random effects model with participants as the random effect factor, and time (with 6 levels) as fixed effect factors based on a restricted maximum likelihood (REML) model. This statistical model will hold all between-time comparisons for all assessment points up to 1095 days from baseline (incl. baseline) and thus allows for evaluation of the average effect, as well as the trajectory over time from baseline to 1095 days follow-up.

**Missing data and Outliers**

The detection of outliers can be an important problem in model building, inference, and analysis of a regression model like the linear mixed models we will apply. Since the presence of outliers can lead to biased estimation, misspecification of the model, and inappropriate predictions. We will apply model diagnostics (incl. ‘Cooks D’), in order to evaluate the distribution of studentized residuals and detect the presence of outliers and influential points; using studentized residuals and Cooks distance for detecting outliers in Y-direction plotted against the predicted estimates.

**Additional analyses**

Non

**Harms**

Any adverse events during or after the treatment in the trial will be noted and categorised into severity.

**Statistical software**

All statistical analyses will be performed in SAS and/or R-studio.

# **Supplementary Figure 1. Flow diagram illustrating the trial flow**

Assessed for eligibility

(n = 18)

Follow-up day 5

(n = 10)

Follow-up day 1

(n = 10)

Treated

(n = 10)

Follow-up day 120

(n = 10)

Excluded (n = 0)

Death (n = 0)

Declined to participate (n = 0)

Lost to follow-up (n = 0)

Analysed

(n = 10)

Follow-up day 1095

(n = 10)

Follow-up day 30

(n = 10)

Excluded (n = 8)

Not meeting inclusion criteria (n = 6)

Declined to participate (n = 1)

Other (n = 1)

**Supplementary table 1. Baseline characteristics of patients**

Previously published in Lynggaard CD, Grønhøj C, Christensen R, et al. Intraglandular Off-the-Shelf Allogeneic Mesenchymal Stem Cell Treatment in Patients with Radiation-Induced Xerostomia: A Safety Study (MESRIX-II). *Stem Cells Transl Med*. 2022;11(5):478-489. doi:10.1093/STCLTM/SZAC011 [3]

| Variable | |  | Patients  (n = 10) |
| --- | --- | --- | --- |
| Age, years | |  | 61.1 (7.5) |
| Male sex, no. (%) | |  | 7 (70 %) |
| *Previous smoking history, no. (%):* | |  |  |
|  | 0 pack years | | 7 (70 %) |
|  | 1-10 pack years | | 1 (10 %) |
|  | >10 pack years | | 2 (20 %) |
| *Primary tumor location, no. (%)* | |  |  |
|  | Tonsil | | 10 (100 %) |
|  | Base of tongue | | 0 |
| p16 positive, no. (%) | |  | 10 (100 %) |
| *UICC8 cancer stage, no (%)* | |  |  |
|  | 1 | | 7 (70 %) |
|  | 2 | | 3 (30 %) |
| Duration since radiotherapy to intervention (years) | | | 5.5 (2.2) |
| *Mean radiation dose, Gy* | |  |  |
|  | Right submandibular gland | | 55.5 (12.9) |
|  | Left submandibular gland | | 56.0 (16.8) |
|  | Right parotid gland | | 28.7 (12.3) |
|  | Left parotid gland | | 25.6 (20.6) |
| Received adjuvant chemotherapy, no. (%) | | | 10 (100 %) |
| DMSO, 10%, no. (%) | |  | 5 (50 %) |
| Treatment over 2 days, no. (%) | |  | 4 (40 %) |
| *Saliva flow rate, mL/min:* | |  |  |
|  | Unstimulated whole saliva flow rate | | 0.13 (0.06) |
|  | Stimulated whole saliva flow rate | | 0.66 (0.34) |
| *EORTC QLQ-H&N-35 (0-100)*: | |  |  |
|  | HNDR | | 73.3 (30.6) |
|  | HNSS | | 46.7 (32.2) |
|  | HNSW | | 26.7 (20.7) |
| XQ (0-100) | |  | 53.5 (23.2) |
| Preformed DSA, no. (%) | |  | 1 (10 %) |

Values are Mean (SD) unless otherwise indicated. Abbreviation: DMSO: Dimethyl sulfoxide, EORTC QLQ-H&N35: the European Organization for Research and Treatment of Cancer Quality of Life Questionnaire, Head and Neck Module; HNDR: domains for dry mouth (HNDR); HNSS: domains for sticky saliva; HNSW: domains for swallowing; XQ: xerostomia Questionnaire; DSA: Donor-specific antibodies

# **Reference**

1 Wolff A, Joshi RK, Ekström J, et al. A Guide to Medications Inducing Salivary Gland Dysfunction, Xerostomia, and Subjective Sialorrhea: A Systematic Review Sponsored by the World Workshop on Oral Medicine VI. Drugs R D 2017;17:1–28.

2 Danish Health and Medicines Authority. Health promotion packages – introduction and recommendations. 2013.

3 Lynggaard CD, Grønhøj C, Christensen R, et al. Intraglandular Off-the-Shelf Allogeneic Mesenchymal Stem Cell Treatment in Patients with Radiation-Induced Xerostomia: A Safety Study (MESRIX-II). Stem Cells Transl Med 2022;11:478–489.
